# Outcomes of Laparoscopic Total Gastrectomy Combined With Spleen-Preserving Hilar Lymphadenectomy for Locally Advanced Proximal Gastric Cancer: A Nonrandomized Clinical Trial

**DOI:** 10.1001/jamanetworkopen.2021.39992

**Published:** 2021-12-20

**Authors:** Chaohui Zheng, Yanchang Xu, Gang Zhao, Lisheng Cai, Guoxin Li, Zekuan Xu, Su Yan, Zuguang Wu, Fangqin Xue, Yihong Sun, Dongbo Xu, Wenbin Zhang, Jin Wan, Peiwu Yu, Jiankun Hu, Xiangqian Su, Jiafu Ji, Ziyu Li, Jun You, Yong Li, Lin Fan, Junpeng Lin, Jianxian Lin, Ping Li, Changming Huang

**Affiliations:** 1 Department of Gastric Surgery, Fujian Medical University Union Hospital, Fuzhou, China; 2 Fujian Medicine University, Teaching Hospital, The First Hospital of PuTian City, Putian, China; 3 Department of Gastrointestinal Surgery, Renji Hospital, Shanghai Jiao Tong University School of Medicine, Shanghai, China; 4 Department of General Surgery Unit 4, Zhangzhou Affiliated Hospital of Fujian Medical University, Zhangzhou, Fujian, China; 5 Department of General Surgery, Nanfang Hospital, Southern Medical University, Guangzhou, China; 6 Department of General Surgery, Jiangsu Province Hospital, Nanjing Medical University, Nanjing, China; 7 Department of Gastrointestinal Surgery, Affiliated Hospital of Qinghai University, Qinghai Medical University, Xining, China; 8 Department of General Surgery Unit 2, Meizhou People's Hospital of Guangdong, Meizhou, China; 9 Department of Gastrointestinal Surgery, Fujian Provincial Hospital, Fuzhou, China; 10 Department of General Surgery, Zhongshan Hospital, Fudan University, Shanghai, China; 11 Department of General Surgery, Longyan First Hospital, Longyan, China; 12 Department of General Surgery, The First Affiliated Hospital of Xinjiang Medical University, Xinjiang Medical University, Wulumuqi, China; 13 Department of General Surgery, Guangdong Provincial Hospital of Chinese Medicine, Guangzhou University of Traditional Chinese Medicine, Guangzhou, China; 14 Department of General Surgery, Southwest Hospital, Third Military Medical University, Chongqing, China; 15 Department of Gastrointestinal Surgery, West China Hospital of Sichuan University, Sichuan University, Chengdu, China; 16 Gastrointestinal Cancer Center, Beijing University Cancer Hospital, Beijing, China; 17 Department of Gastrointestinal Surgery, The First Affiliated Hospital of Xiamen University, Xiamen University, Xiamen, China; 18 Department of General Surgery, Guangdong Provincial People's Hospital, Guangzhou, China; 19 Department of General Surgery, The First Affiliated Hospital of Xi'an Jiaotong University, Xi’an, China

## Abstract

**Importance:**

The long-term survival of patients with laparoscopic total gastrectomy combined with spleen-preserving splenic hilar lymphadenectomy (LSTG) for advanced upper-third gastric cancer (AUTGC) and the association of splenic hilar lymph node (LN-10) metastasis with survival remain controversial.

**Objective:**

To evaluate the long-term outcomes of LSTG and the value index of LN-10 metastasis for patients with AUTGC.

**Design, Setting, and Participants:**

The Chinese Laparoscopic Gastrointestinal Surgery Study 4 (CLASS-04) was a prospective, multicenter, single-arm trial that involved 19 centers in China. A total of 251 eligible patients with clinical stage T2, T3, or T4a upper-third gastric cancer without distant metastases were enrolled from September 1, 2016, to October 31, 2017. The final follow-up was on December 31, 2020.

**Interventions:**

All patients were enrolled to undergo LSTG.

**Main Outcomes and Measures:**

The main outcomes were the 3-year overall survival (OS) and disease-free survival (DFS). Multivariate analyses were used to explore the association of LN-10 metastasis with survival.

**Results:**

Among the 251 patients, 246 (98.0%; mean [SD] age, 60.1 [9.4] years; 197 [80.1%] male) underwent LSTG and completed the study. The 3-year OS was 79.1% (95% CI, 74.0%-84.2%), and the 3-year DFS was 73.1% (95% CI, 67.4%-78.8%). In addition, the 3-year therapeutic value index of LN-10 dissection was 4.5, exceeding the indexes for the partial D2 LN group (including LNs 5, 6, 11d, and 12a). Nineteen patients (7.7%) with LN-10 metastasis had significantly worse survival than the nonmetastasis group, and multivariate analysis revealed that splenic LN-10 metastasis was an independent risk factor (OS: hazard ratio [HR], 2.38; 95% CI, 1.08-5.26; *P* = .03; DFS: HR, 2.28; 95% CI, 1.12-4.63; *P* = .02). Moreover, patients with LN-10 metastasis were more likely to have recurrence (42.1% vs 20.7%, *P* = .03), especially when multiple site metastasis was present (21.1% vs 4.4%, *P* = .01). However, patients with LN-10 metastasis who received adjuvant chemotherapy had significantly better OS and DFS than those without adjuvant chemotherapy and achieved the same oncologic effect as those without LN-10 metastasis.

**Conclusions and Relevance:**

This results of this study suggest that LSTG for AUTGC has feasible long-term outcomes. In addition, patients with LN-10 metastasis may have worse survival and may be more prone to recurrence.

## Key Points

**Question:**

What are the 3-year outcomes after laparoscopic total gastrectomy combined with spleen-preserving splenic hilar lymphadenectomy for patients with locally advanced proximal gastric cancer?

**Findings:**

In this nonrandomized clinical trial of 251 patients with clinically staged, locally advanced upper-third gastric cancer, the 3-year overall survival was 79.1%. The 3-year therapeutic value index of lymph node 10 dissection was 4.5, exceeding the index for the partial D2 lymph node group.

**Meaning:**

The findings of this study suggest the utility of laparoscopic total gastrectomy combined with spleen-preserving splenic hilar lymphadenectomy for clinically staged, locally advanced upper-third gastric cancer.

## Teaser

This nonrandomized clinical trial of patients with advanced upper-third gastric cancer assesses the outcomes of patients with laparoscopic total gastrectomy combined with spleen-preserving splenic hilar lymphadenectomy and the association of splenic hilar lymph node metastasis with survival.

## Introduction

Gastric cancer is a common cancer and 1 of the leading causes of cancer-related deaths worldwide.^1^ Gastric cancer tends to migrate proximally, and the incidence of proximal gastric cancer has increased since 1976.^2,3^ The standard surgical treatment to advanced upper-third gastric cancer (AUTGC) recommended by the Japanese Gastric Cancer Association is total gastrectomy with D2 lymphadenectomy.^4,5^ However, whether the splenic hilar lymph node (LN-10) should be included in the scope of D2 LN dissection^4,5^ and whether LN-10 metastasis affects the outcomes of gastric cancer is still controversial.^6,7^ Although previous prospective studies^8,9,10^ have found that LN-10 dissection increased postoperative morbidity without improving survival, these randomized clinical trials all included a splenectomy to accomplish LN-10 dissection. Because the spleen is an important immune organ and the LNs of the splenic hilum are located deep and close to splenic vessels, the operation is difficult, so these studies^8,9,10^ tend to preserve the spleen. The safety and feasibility of laparoscopic techniques in gastric cancer have been increasingly confirmed.^11,12^ Although previous studies^13,14,15^ have found that laparoscopic spleen-preserving splenic hilar lymphadenectomy is technically feasible, most are single-center studies,^13,14,15^ and high-level clinical evidence is lacking.

To our knowledge, the Chinese Laparoscopic Gastrointestinal Surgery Study 4 (CLASS-04) was the first large-scale, multicenter prospective, trial designed to provide evidence of the surgical and oncologic safety of laparoscopic total gastrectomy combined with spleen-preserving splenic hilar lymphadenectomy (LSTG) for AUTGC clinical stage cT2-4a, N^−/+^, M0). A previous study^16^ reported that LSTG for AUTGC was safe and effective when performed by experienced surgeons. This study aimed to assess the oncologic outcomes of LSTG and the association of LN-10 metastasis with survival for AUTGC.

## Methods

### Study Design

The CLASS-04 study was a multicenter, nonrandomized, single-arm, prospective clinical trial conducted at 19 tertiary hospitals in China from September 1, 2016, to October 31, 2017. The final follow-up was on December 31, 2020. The informed consent form and study protocol were approved by the institutional review board of each participating hospital, and all patients provided written informed consent. All data were deidentified. An independent data and safety monitoring committee monitored the trial safety and progress. This study followed the Transparent Reporting of Evaluations With Nonrandomized Designs (TREND) reporting guideline.

### Study Patients

The trial enrolled patients with gastric cancer suitable for undergoing LSTG for curative intent. Patients were included if they were 18 to 75 years of age; had an American Society of Anesthesiology score of class I, II, or III; had histologically confirmed gastric adenocarcinoma with a clinical stage of cT2-4a, N^−/+^, M0 according to the seventh edition of the American Joint Committee on Cancer’s (AJCC’s) *Cancer Staging Manual*^17^; had tumors located in the upper third of the stomach by preoperative evaluation; and provided written informed consent before participation. Patients were excluded if they had enlarged splenic hilar LNs with integration into a mass and surrounding the blood vessels or if they had clinical T4b tumors and underwent preoperative neoadjuvant therapy. Detailed eligibility criteria are listed in eTable 1 in the Supplement.

### Surgical Quality Control, Procedures, and Follow-up

The detailed criteria for the participating surgeons and surgical quality control in CLASS-04 have been described previously.^16^ In brief, the surgeons were required to have performed 50 LSTGs before the trial. A series of photographs obtained during surgery and an unedited video of the laparoscopic operation were required to be submitted to the CLASS data center within 1 week after surgery. Detailed surgical quality controls are shown in eFigure 1 in the Supplement. The CLASS Research Committee monitored and reviewed these materials regularly to ensure continuing quality during surgery.

Laparoscopic total gastrectomy with D2 lymphadenectomy was performed according to the Japanese Gastric Cancer Treatment Guidelines.^18^ Laparoscopic spleen-preserving LN-10 dissection was performed. For patients with pathologic stage II or higher tumors,^17^ adjuvant chemotherapy with 6 months of fluorouracil-based chemotherapy was recommended, with the choice of regimen and treatment duration at the discretion of the treating oncologist.

A minimum follow-up of 36 months was required and achieved for each patient after surgery. Follow-up was conducted every 3 months for the first 2 years postoperatively and every 6 months for the next 3 years. Most routine follow-up appointments included a physical examination, laboratory testing (including CA19-9, CA72-4, and carcinoembryonic antigen measurements), chest radiography, and abdominopelvic ultrasonography or computed tomography, along with an annual endoscopic examination.

### End Points and Definitions

The primary end point of this trial was overall postoperative morbidity rate, which has been previously reported.^16^ The 3-year overall survival (OS) and disease-free survival (DFS) were measured as secondary end points, which were the main outcomes in this study. The OS was calculated from the date of surgery to the date of last contact or death. The DFS was defined as the interval from the date of the operation to the recurrence of gastric cancer or death.^19^ Recurrence was identified by medical history and physical examination in combination with imaging evaluation, cytologic analysis, or tissue biopsy (preferred when feasible).^11^ To evaluate the therapeutic value of dissection at each LN station, we used the therapeutic value index proposed by Sasako et al.^20^ The frequency of metastasis to each station was then calculated by dividing the number of patients with metastasis at that station by the number in whom the station was dissected. The 3-year therapeutic value index was calculated by multiplying the frequency of metastasis to the station by the 3-year survival rate of patients with metastasis to each station. Postoperative complications were limited to events that occurred within 90 days after surgery. Early and late complications were regarded as the occurrence of events within and beyond 30 days after surgery, respectively.^21^ Surgical mortality was defined as any death that occurred during the hospital stay or any death associated with surgical complications within the first 90 postoperative days.^22^ The staging in this study was performed according to the seventh edition of the AJCC’s *Cancer Staging Manual*.^17^

### Statistical Analysis

The sample size calculation was conducted using nQuery Advisor, version 7.0 (Statistical Solutions Ltd), which has been described previously.^16^ Continuous variables were described as the means (SDs) or medians (IQRs), and categorical variables were described as numbers (percentages). The 3-year DFS and OS rates were calculated using the Kaplan-Meier method, and the log rank test was used to assess differences between tumor stages. A multivariable Cox proportional hazards regression model was used to identify the independent factors associated with survival. All tests were 2-sided, and statistical significance was inferred at *P* < .05. Statistical analyses were performed using SPSS, version 22.0 (IBM Inc).

## Results

### Patient Demographic Characteristics

**Figure 1.**
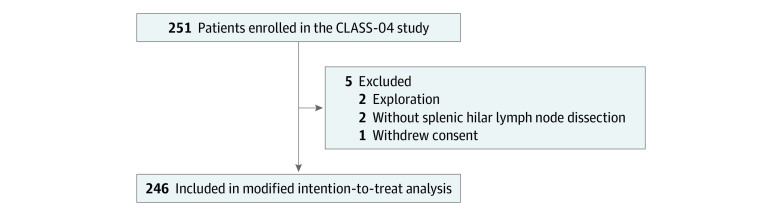
Flow of Patient Enrollment CLASS indicates Chinese Laparoscopic Gastrointestinal Surgery Study.

A total of 246 patients (mean [SD] age, 60.1 [9.4] years; 197 [80.1%] male) who underwent LSTG were included in the modified intention-to-treat analysis. Five patients were excluded, including 2 patients who underwent exploratory surgery, 1 patient who withdrew informed consent, and 2 patients who did not undergo LN-10 dissection. The baseline characteristics of the patients are given in Table 1, and the flow diagram is provided in Figure 1. With regard to clinical TNM stage, 14 patients (5.7%) had clinical stage I tumors, 104 (42.3%) had clinical stage II tumors, and 128 (52.0%) had clinical stage III tumors at primary diagnosis.

**Table 1.** Patient Baseline Clinicopathologic Characteristics^a^

Characteristic	Finding (N = 246)
Age, mean (SD), y	60.1 (9.4)
Sex	
Male	197 (80.1)
Female	49 (19.9)
BMI, mean (SD)	22.4 (2.8)
ECOG performance status	
0	163 (66.3)
1	83 (33.7)
Comorbidity	
0	192 (78.0)
≥1	54 (22.0)
Clinical T stage	
T2	27 (11.0)
T3	96 (39.0)
T4a	123 (50.0)
Clinical N stage	
N0	80 (32.5)
N^+^	166 (67.5)
Clinical TNM stage	
I	14 (5.7)
II	104 (42.3)
III	128 (52.0)
No. of lymph nodes, mean (SD)	
Retrieved	42.7 (16.2)
Metastatic	4.9 (7.1)
Lymph node 10 metastasis	19 (7.7)
Tumor size, mean (SD), cm	4.3 (2.2)
Histologic findings	
SRC or poorly differentiated AC	144 (58.5)
Other	102 (41.5)
Cross-sectional part	
Nongreater curvature	223 (90.7)
Greater curvature	23 (9.3)
Invasion	
Vascular	95 (38.6)
Lymphatic	66 (26.8)
Perineural	141 (57.3)
Pathologic T stage	
T1	19 (7.7)
T2	33 (13.4)
T3	85 (34.6)
T4a	108 (43.9)
T4b	1 (0.4)
Pathologic N stage	
N0	84 (34.1)
N1	46 (18.7)
N2	49 (19.9)
N3	67 (27.2)
Pathologic TNM stage	
I	39 (15.9)
II	78 (31.7)
III	129 (52.4)
Received adjuvant chemotherapy	182 (74.0)

Abbreviations: AC, adenocarcinoma; BMI, body mass index (calculated as weight in kilograms divided by height in meters squared); ECOG, Eastern Cooperative Oncology Group; SRC, signet ring cell.

^a^ Data are presented as number (percentage) of patients unless otherwise indicated.

### Pathologic Outcomes and Complications

The postoperative characteristics of the patients are given in Table 1. The mean (SD) number of retrieved LNs was 42.1 (16.2), and 19 patients had LN-10 metastasis. The incidence of LN-10 metastasis (7.7%) was higher than that of LN-5 (3.7%), LN-6 (4.5%), LN-11d (6.5%), and LN-12a (1.6%) metastasis (eFigure 2A in the Supplement). A total of 223 patients (90.7%) had tumors located on the nongreater curvature, and 182 patients (74.0%) received adjuvant chemotherapy after surgery.

Because there were minor differences between patients included in this analysis and those in the previous study^16^ and we did not report late complications, we further clarified the postoperative complications in detail. The incidence of early complications was 14.2%, of which local complications constituted 8.5% and systemic complications 6.9% (eTable 2 in the Supplement). Four patients (1.6%) experienced late complications, including 2 with intestinal obstruction, 1 with anastomotic stenosis, and 1 with malabsorption. The incidence of Clavien-Dindo grade III or higher complications was 3.7%, and 1 patient died of liver and kidney failure within 90 days after the operation.

### Survival Outcomes and Benefits

**Figure 2.**
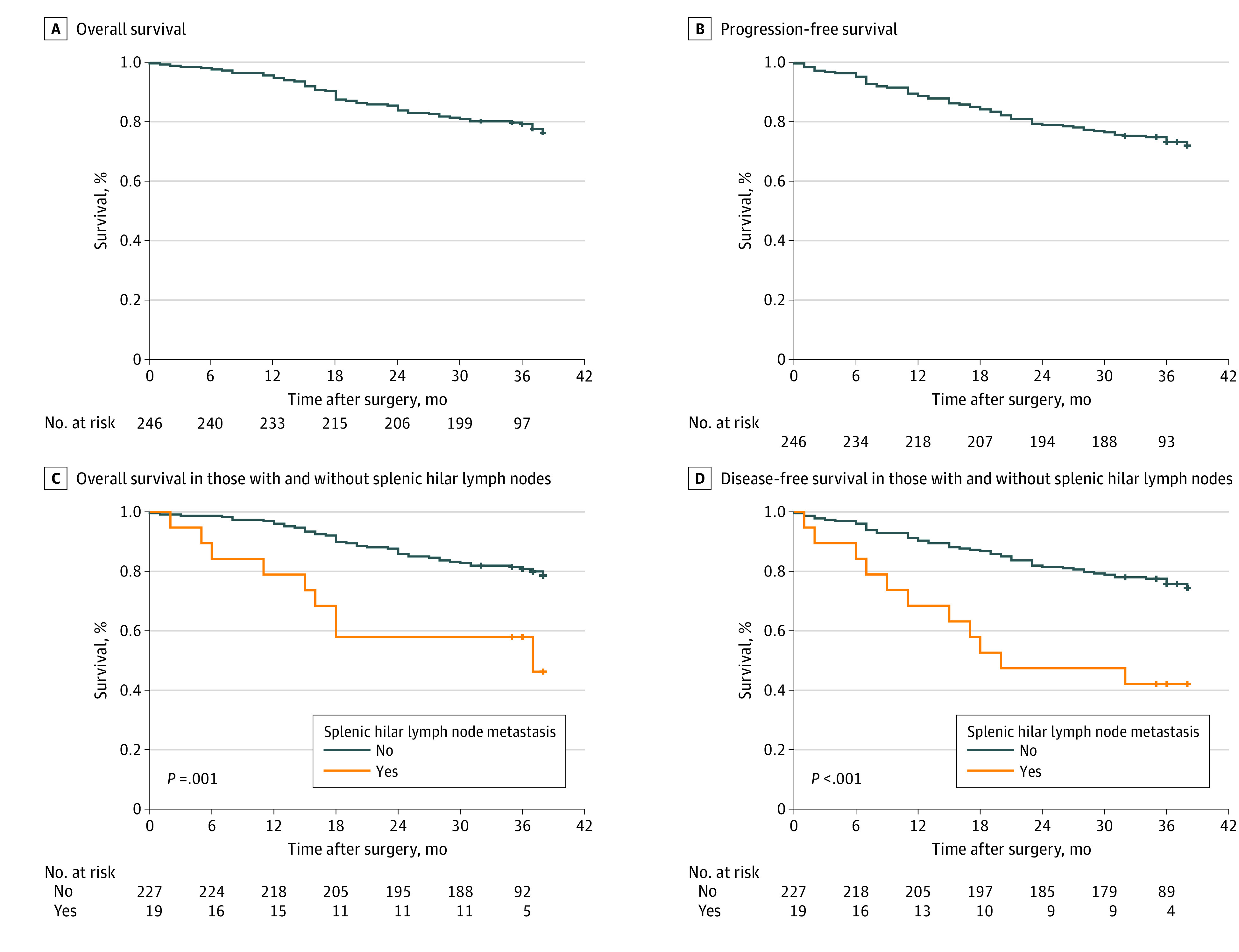
Overall and Disease-Free Survival

After a median follow-up time of 36.0 months (IQR, 35.0-37.3 months), 45 patients died. For all patients, the 3-year OS rate was 79.1% (95% CI, 74.0%-84.2%) and the 3-year DFS rate was 73.1% (95% CI, 67.4%-78.8%) (Figure 2A-B). The 3-year OS rates were 97.4% for patients with pathologic stage I disease, 89.0% for those with stage II disease, and 67.4% for those with stage III disease. The 3-year DFS rates were 97.4% for patients with pathologic stage I disease, 84.5% for those with stage II disease, and 58.8% for those with stage III disease. These findings were similar to those of patients who underwent distal gastrectomy with standard D2 lymphadenectomy (including open and laparoscopic surgery) in a multicenter prospective study (CLASS-01)^11^ (eFigure 3 in the Supplement).

Recurrence was found in 55 patients. Peritoneal recurrence was the most common site (7.3%), whereas the incidence of local recurrence was 3.3%, the incidence of distant metastasis was 4.1%, and the incidence of multiple metastasis was 5.7% (Table 2). The 3-year therapeutic value index of regional LN dissection is shown in eFigure 2B in the Supplement. The 3-year index of LN-10 dissection was 4.5, which was higher than that of the partial D2 LN group, including LN-5 (2.1), LN-6 (2.5), LN-11d (4.1), and LN-12a (0.4) dissections.

**Table 2.** Pattern of First Tumor Recurrence

Event	No. (%) of patients			*P* value
	Total (N = 246)	LN-10		
		No (n = 227)	Yes (n = 19)	
Any recurrence	55 (22.4)	47 (20.7)	8 (42.1)	.03
Local	8 (3.3)	8 (3.5)	0 (0.0)	
Anastomosis	7 (2.8)	7 (3.1)	0 (0.0)	>.99
Regional lymph node	1 (0.4)	1 (0.4)	0 (0.0)	
Peritoneum	18 (7.3)	15 (6.6)	3 (15.8)	.31
Distant	10 (4.1)	9 (4.0)	1 (5.3)	
Liver	2 (0.8)	2 (0.9)	0 (0.0)	>.99
Lung	2 (0.8)	2 (0.9)	0 (0.0)	
Bone	1 (0.4)	1 (0.4)	0 (0.0)	
Brain	1 (0.4)	1 (0.4)	0 (0.0)	
Pancreas	1 (0.4)	0	1 (5.3)	
Adrenal gland	1 (0.4)	1 (0.4)	0	
Distant lymph node	2 (0.8)	2 (0.9)	0	
Sites				
Multiple	14 (5.7)	10 (4.4)	4 (21.1)	.01
Uncertain	5 (2.0)	5 (2.2)	0	>.99

### Association of LN-10 Metastasis With Outcomes

The 3-year OS and DFS for LN-10 metastasis were significantly lower than those of LN-10 nonmetastasis (3-year OS, 63.2% vs 80.4%; *P* = .001; 3-year DFS, 47.4% vs 75.3%; *P* < .001) (Figure 2C-2D). Univariate analysis found that LN-10 metastasis was associated with poor survival (OS: hazard ratio [HR], 3.06; 95% CI, 1.50-6.26; *P* = .002; DFS: HR, 2.70; 95% CI, 1.38-5.29; *P* = .004) (eTable 3 in the Supplement). Further multivariable analysis revealed that LN-10 metastasis was an independent risk factor for OS (HR, 2.38; 95% CI, 1.08-5.25; *P* = .03) and DFS (HR, 2.28; 95% CI, 1.12-4.63; *P* = .01). Comorbidities (≥1) were also an independent risk factor for OS (HR, 2.58; 95% CI, 1.45-4.58; *P* < .001) and DFS (HR, 2.35; 95% CI, 1.39-3.96; *P* = .001), as were pathologic TNM stage III (OS: HR, 3.95; 95% CI, 1.91-8.18; *P* < .001; DFS: HR, 3.47; 95% CI, 1.83-6.56; *P* < .001) and adjuvant chemotherapy (OS: HR, 0.29; 95% CI, 0.16-0.53; *P* < .001; DFS: HR, 0.37; 95% CI, 0.21-0.64; *P* < .001).

The recurrence rate in the LN-10 metastasis group at 3 years after surgery was significantly higher than that in the LN-10 nonmetastasis group (42.1% vs 20.7%; *P* = .03), especially in the multiple metastasis group (21.1% vs 4.4%; *P* = .01). However, in other recurrence patterns, both groups were similar (Table 2).

### Adjuvant Chemotherapy for LN-10 Metastasis

A total of 19 patients (mean [SD] age, 57.3 [11.1] years; 16 [84.2%] male) had LN-10 metastasis. A total of 17 patients had tumors located on the nongreater curvature, and 2 had tumors located on the greater curvature. Sixteen patients (84.2%) had signet ring cell carcinoma or poorly differentiated adenocarcinoma. In terms of staging, 16 patients had clinical stage II tumors, and 3 patients had clinical stage III tumors. Among these patients, 14 (73.7%) received adjuvant chemotherapy, and 5 (26.3%) refused further treatment. eTable 4 in the Supplement indicates that the characteristics were well balanced between the 2 groups. The long-term survival of patients with LN-10 metastasis who received adjuvant chemotherapy was significantly better than that of patients without adjuvant chemotherapy (OS: HR, 0.07; 95% CI, 0.01-0.37; *P* = .002; DFS: HR, 0.11; 95% CI, 0.03-0.47; *P* = .003) (eFigure 4A-B in the Supplement) and was comparable to that of patients without LN-10 metastasis (OS: HR, 1.05; 95% CI, 0.33-3.37; *P* = .94; DFS: HR, 1.50; 95% CI, 0.60-3.74; *P* = .39).

## Discussion

This multicenter, prospective nonrandomized clinical trial conducted at 19 centers in China found that LSTG for patients with AUTGC clinical stage (cT2-4a, N^−/+^, M0) has feasible long-term survival benefits. The 3-year OS rate was 79.1%, the 3-year DFS rate was 73.1%, and the 3-year therapeutic value index of LN-10 dissection was better than that of the partial D2 LN group (including LN-5, LN-6, LN-11d, and LN-12a dissections). In addition, LN-10 metastasis was an independent risk factor; however, postoperative adjuvant chemotherapy could improve the long-term outcome of patients with LN-10 metastasis.

The incidence of proximal gastric cancer has continued to increase during the past few decades,^3^ and the metastatic rate of LN-10 is as high as 8.8% to 20.9% in proximal gastric cancer.^23,24^ Therefore, previous guidelines recommended standard D2 LN lymphadenectomy, including LN-10 lymphadenectomy, for locally advanced proximal gastric cancer.^4,18^ However, the recent treatment guideline of the 15th Japanese Gastric Cancer Association did not recommend splenectomy for removal of the LN-10 in D2 lymphadenectomy during total gastrectomy based on the result of the Japanese Clinical Oncology Group 0110 trial.^5,9^ Participants in this trial underwent splenectomy to remove the LN-10, and the incidence of early postoperative complications was as high as 30.3%,^9^ but it was only 14.2% in our study. This result may be attributable to spleen-preserving lymphadenectomy and the greatly reduced surgical trauma; laparoscopic approaches have benefits over open operations through visual magnification, better exposure, and more delicate maneuvers around organs, vessels, and nerves.^11^

Currently, whether LN-10 dissection has survival benefits remains controversial.^7,9^ According to the Japanese gastric cancer treatment guidelines, distal gastrectomy with D2 LN dissection is the current standard for advanced gastric cancer located in the lower or middle third of the stomach.^5^ Compared with the long-term survival of patients who underwent standard radical gastrectomy, we found that the survival of patients with different stages of disease who underwent LSTG was comparable to that of patients with laparoscopic distal gastrectomy and open distal gastrectomy in the CLASS-01 study,^11^ which indicates that LSTG for AUTGC has feasible long-term outcomes. Moreover, in this CLASS-04 study, we found that the metastasis rate and 3-year therapeutic value index of LN-10 dissection was higher than that of partial D2 LN stations dissections. Therefore, whether LN-10 should be included in D2 LN dissection for patients with locally advanced upper gastric cancer needs to be confirmed by more prospective, multicenter randomized clinical studies.

Whether LN-10 metastasis affects the survival of patients with gastric cancer is still controversial. Shin et al^6^ found that the 5-year OS of the hilar node metastasis group was only 11.0%, which was significantly lower than that of the nonmetastasis group (51.6%). However, another study^7^ found that there was no significant difference in survival between patients with splenic hilar metastasis (51.3%) and those without such metastasis (42.1%). In the current trial, the LN-10 metastasis group did not reach the median OS time and median DFS time. In addition, the median DFS time in the LN-10 nonmetastasis group was 32 months, but this group did not reach the median overall survival time. However, in patients with splenic hilar metastasis, the 3-year OS was 17.2% and the 3-year DFS was 27.9% less than the rates in patients without metastasis. Furthermore, we confirmed that LN-10 metastasis was an independent risk factor, and the risk of death or recurrence in patients with LN-10 metastasis was more than twice that in those without LN-10 metastasis. In addition, patients with LN-10 metastasis were more likely to have multiple-site metastasis. Thus, systemic follow-up should be strengthened for patients with LN-10 metastasis to find recurrence early.

Adjuvant chemotherapy after surgery is a standard component of therapies for patients with resectable advanced gastric cancer.^25^ Our study confirmed that adjuvant chemotherapy was an independent protective factor for AUTGC. However, because of the sensitivity and toxicity of chemotherapy, not all patients can benefit from adjuvant chemotherapy,^25,26^ and whether adjuvant chemotherapy can improve the survival of patients with LN-10 metastasis has not been confirmed. In this study, in patients with LN-10 metastasis who received adjuvant chemotherapy, the 3-year OS was 71.4% and the 3-year DFS was 43.7%, whereas those without adjuvant chemotherapy all died within 3 years after surgery. In addition, no significant difference was found in clinicopathologic factors between patients with and without adjuvant chemotherapy in the LN-10 metastasis group. Therefore, for patients with LN-10 metastasis, we recommend adjuvant chemotherapy to improve long-term survival.

### Limitations

This trial has several limitations. First, as a single-arm trial, patients with LN-10 dissection and those without LN-10 dissection were not compared. However, our findings can be used as preliminary results and an evidence-based basis for future prospective randomized clinical trials. Second, only 19 patients had LN-10 metastasis in this study, which may cause bias because of the small sample size. This phenomenon may be attributable to the fact that most of the tumors were located on the nongreater curvature. Despite this, the index of LN-10 dissection was better than partial D2 LN dissection, which provided important evidence for the application of LN-10 dissection in AUTGC. Finally, our results might not be generalizable to less experienced surgeons. However, considering the survival improvements after gastrectomy by centralization, LSTG for AUTGC performed by a surgeon specializing in gastric cancer surgery is ideal.

## Conclusions

The findings of this study suggest that laparoscopic total gastrectomy combined with spleen-preserving splenic hilar lymphadenectomy for AUTGC has promising long-term outcomes and therapeutic benefits. Furthermore, patients with LN-10 metastasis may have worse survival and may be more prone to recurrence.

## Notes

Supplement. **eTable 1.** Eligibility Criteria of the Trial **eTable 2.** Distribution of Early and Late Complications **eTable 3.** Univariable Analysis for Predicting Overall Survival and Disease-Free Survival **eTable 4.** Clinical and Pathologic Characteristics Between Patients With and Without Adjuvant Chemotherapy **eFigure 1.** Surgical Quality Control for CLASS-04 Trial (A) and Field of Lymph Node Dissection (B-G) **eFigure 2.** The Incidence of Lymph Node (LN) Metastasis (A) and 3-Year Therapeutic Value Index (B) of LN Dissection **eFigure 3.** Comparison of the Long-term Survival of Patients With Different Stages in the CLASS-04 and CLASS-01 Studies **eFigure 4.** Kaplan-Meier Curves of Overall Survival (A) and Disease-Free Survival (B) for Patients With No. 10 Lymph Node (LN) Metastasis and Without Metastasis Stratified by Adjuvant Chemotherapy
