# Supplementary material for: Outcomes of Laparoscopic Total Gastrectomy Combined With Spleen-Preserving Hilar Lymphadenectomy for Locally Advanced Proximal Gastric Cancer: A Nonrandomized Clinical Trial
Source: JAMA Netw Open. 2021 Dec 20;4(12):e2139992. doi: 10.1001/jamanetworkopen.2021.39992 (PMC8689389; doi:10.1001/jamanetworkopen.2021.39992)
Supplement: Supplement. — eTable 1. Eligibility Criteria of the Trial eTable 2. Distribution of Early and Late Complications eTable 3. Univariable Analysis for Predicting Overall Survival and Disease-Free Survival eTable 4. Clinical and Pathologic Characteristics Between Patients With and Without Adjuvant Chemotherapy eFigure 1. Surgical Quality Control for CLASS-04 Trial (A) and Field of Lymph Node Dissection (B-G) eFigure 2. The Incidence of Lymph Node (LN) Metastasis (A) and 3-Year Therapeutic Value Index (B) of LN Dissection eFigure 3. Comparison of the Long-term Survival of Patients With Different Stages in the CLASS-04 and CLASS-01 Studies eFigure 4. Kaplan-Meier Curves of Overall Survival (A) and Disease-Free Survival (B) for Patients With No. 10 Lymph Node (LN) Metastasis and Without Metastasis Stratified by Adjuvant Chemotherapy [file jamanetwopen-e2139992-s001.pdf]

Supplementary Online Content

Zheng C, Xu Y, Zhao G, et al. Outcomes of laparoscopic total gastrectomy combined with spleen-preserving hilar lymphadenectomy for locally advanced proximal gastric cancer: a nonrandomized clinical trial. *JAMA Netw Open*. 2021;4(12):e2139992. doi:10.1001/jamanetworkopen.2021.39992

eTable 1. Eligibility Criteria of the Trial

eTable 2. Distribution of Early and Late Complications

eTable 3. Univariable Analysis for Predicting Overall Survival and Disease-Free Survival

eTable 4. Clinical and Pathologic Characteristics Between Patients With and Without Adjuvant Chemotherapy

eFigure 1. Surgical Quality Control for CLASS-04 Trial (A) and Field of Lymph Node Dissection (B-G)

eFigure 2. The Incidence of Lymph Node (LN) Metastasis (A) and 3-Year Therapeutic Value Index (B) of LN Dissection

eFigure 3. Comparison of the Long-term Survival of Patients With Different Stages in the CLASS-04 and CLASS-01 Studies

eFigure 4. Kaplan-Meier Curves of Overall Survival (A) and Disease-Free Survival (B) for Patients With No. 10 Lymph Node (LN) Metastasis and Without Metastasis Stratified by Adjuvant Chemotherapy

This supplementary material has been provided by the authors to give readers additional information about their work.

© 2021 Zheng C et al. *JAMA Network Open.*

eTable 1. Eligibility Criteria of the Trial

Inclusion Criteria
(1) Age between 18 and 75 years
(2) Advanced upper third gastric adenocarcinoma (cT2-4a, N-/+, M0) at preoperative evaluation according to the American Joint Committee on Cancer (AJCC) Cancer Staging Manual Seventh Edition
(3) Heart, lungs, kidneys and other vital organs function well, with no obvious surgical contraindications
(4) Preoperative examination with no distant metastasis, no significantly enlarged lymph nodes around abdominal main artery, and tumor not a direct violation of the pancreas, spleen and other surrounding organs
(5) American Society of Anesthesiology (ASA) score class I, II, or III
(6) Written informed consent
Exclusion Criteria:
(1) Women during pregnancy or breast-feeding
(2) Severe mental disorder
(3) History of previous upper abdominal surgery (except laparoscopic cholecystectomy)
(4) Enlarged splenic hilar lymph nodes with integration into a mass and surrounding the blood vessels
(5) History of unstable angina or myocardial infarction within past six months
(6) History of cerebrovascular accident within past six months
(7) History of continuous systematic administration of corticosteroids within one month
(8) History of previous neoadjuvant chemotherapy or radiotherapy
(9) T4b tumors
(10) Emergency surgery due to complication (bleeding, obstruction or perforation) caused by gastric cancer
(11) FEV1(Forced expiratory volume in one second)＜50% of predicted values<br>(12) Peritoneal lavage cytology is positive for cancer cells*
Abbreviations: AJCC, American Joint Committee on Cancer; ASA, American Society of Anesthesiology FEV1, forced expiratory volume in 1 second.

* The peritoneal lavage was performed intraoperatively.

© 2021 Zheng C et al. *JAMA Network Open.*

eTable 2. Distribution of Early and Late Complications

Complication	All Population (n=246)
**Total early complications**	35(14.2%)
**Local complications**	21(8.5%)
Abdominal infection	12(4.9%)
Wound infection	2(0.8%)
Anastomotic leakage	7(2.8%)
Lymphatic leakage	1(0.4%)
Anastomotic bleeding	2(0.8%)
Abdominal bleeding	3(1.2%)
**Systemic complications**	17(6.9%)
Pulmonary	13(5.3%)
Cardiac	2(0.8%)
Renal	3(1.2%)
Hepatic	1(0.4%)
Other	3(1.2%)
**Total late complications**	4(1.6%)
Intestinal obstruction	2(0.8%)
Anastomotic stenosis	1(0.4%)
Malabsorption	1(0.4%)
**Clavien-Dindo complication grade**	
I or II	29(11.8%)
≥ III	9(3.7%)
**90-d mortality**	1(0.4%)

© 2021 Zheng C et al. *JAMA Network Open.*

eTable 3. Univariable Analysis for Predicting Overall Survival and Disease-Free Survival

Clinicopathologic Characteristics	OS			DFS	
	HR (95% CI)	P		HR (95% CI)	P
Age	1.02(0.99-1.05)	.32		1.02(0.99-1.05)	.18
Sex					
Men	Reference			Reference	
Women	0.80(0.43-1.49)	.48		0.79(0.45-1.39)	.41
BMI	0.98(0.89-1.08)	.65		0.99(0.91-1.08)	.81
ECOG					
0	Reference			Reference	
1	1.19(0.69-2.05)	.53		1.05(0.64-1.73)	.85
Comorbidity					
None	Reference			Reference	
One or more	2.73(1.58-4.71)	**<.001**		2.44(1.48-4.01)	**<.001**
Tumor size	1.13(1.03-1.24)	**.007**		1.13(1.05-1.23)	**.002**
Histology					
Other	Reference			Reference	
SRC or poorly differentiated AC	2.49(1.34-4.65)	**.004**		2.17(1.27-3.72)	**.005**
Cross-sectional part					
Non-greater curvature	Reference			Reference	
Greater curvature	2.06(1.01-4.20)	**.05**		1.62(0.80-3.26)	.18
No. of retrieved lymph nodes	0.98(0.97-1.00)	.08		1.00(0.99-1.02)	.84
No.10 lymph node metastasis	3.06(1.50-6.26)	**.002**		2.70(1.38-5.29)	**.004**
Vascular invasion	2.56(1.50-4.37)	**.001**		2.74(1.69-4.44)	**<.001**
Lymphatic invasion	1.86(1.08-3.21)	**.03**		1.84(1.12-3.01)	**.02**
Perineural invasion	1.78(1.01-3.16)	**.05**		1.78(1.06-2.97)	**.03**
Pathologic TNM stage					
I	NA			NA	
II	Reference			Reference	
III	3.55(1.74-7.27)	**.001**		3.32(1.78-6.21)	**<.001**
Received adjuvant chemotherapy	0.64(0.37-1.13)	.13		0.82(0.48-1.40)	.47

Abbreviation: ECOG, Eastern Cooperative Oncology Group score; SRC, Signet-ring cell; AC,

© 2021 Zheng C et al. *JAMA Network Open.*

Adenocarcinoma.

eTable 4. Clinical and Pathologic Characteristics Between Patients With and Without Adjuvant Chemotherapy

Characteristics	No ACT (n=5)	ACT (n=14)	P
Age, y			
Mean (SD)	61.8(15.4)	55.6(9.3)	.30
Sex			.53
Women	0(0.0%)	3(21.4%)	
Men	5(100.0%)	11(78.6%)	
BMI			
Mean (SD)	23.0(3.3)	20.7(2.7)	.14
ECOG			.13
0	5(100.0%)	8(57.1%)	
1	0(0.0%)	6(42.9%)	
Comorbidity			>.99
None	5(100.0%)	13(92.9%)	
One or more	0(0.0%)	1(7.1%)	
Tumor size, cm			
Mean (SD)	6.6(2.6)	4.8(2.4)	.18
Histology			.53
SRC or poorly differentiated AC	5(100.0%)	11(78.6%)	
Other	0(0.0%)	3(21.4%)	
Cross-sectional part			.06
Non-greater curvature	3(60.0%)	14(100.0%)	
Greater curvature	2(40.0%)	0(0.0%)	
Clinical T stage			.62
T2	0(0.0%)	1(7.1%)	
T3	1(20.0%)	5(35.7%)	
T4a	4(80.0%)	8(57.1%)	
Clinical N stage			>.99
N0	1(20.0%)	2(14.3%)	
N+	4(80.0%)	12(85.7%)	
Clinical TNM stage			.93
II	1(20.0%)	5(35.7%)	
III	4(80.0%)	9(64.3%)	
Vascular invasion	4(80.0%)	9(64.3%)	.93
Lymphatic invasion	3(60.0%)	8(57.1%)	>.99
Perineural invasion	5(100.0%)	12(85.7%)	>.99
Pathologic T stage			.17
T2	0(0.0%)	2(14.3%)	
T3	1(20.0%)	7(50.0%)	
T4a	4(80.0%)	5(35.7%)	
Pathologic N stage			.31
N1	0(0.0%)	3(21.4%)	
N2	1(20.0%)	1(7.1%)	
N3	4(80.0%)	10(71.4%)	

© 2021 Zheng C et al. *JAMA Network Open.*

Pathologic TNM stage			.53
II	0(0.0%)	3(21.4%)	
III	5(100.0%)	11(78.6%)	

Abbreviation: ACT, Adjuvant Chemotherapy; ECOG, Eastern Cooperative Oncology Group score; SRC, Signet-ring cell; AC, Adenocarcinoma.

A

Surgeon eligibility

- experience for > 50 cases in LSTG
- the annual no. > 300 cases of gastric cancer surgery in each institute

Video evaluation

- three consecutive unedited videos of LSTG
- two in each were randomly selected
- double-blind peer review
- radicality confirmed by 3 reviewers

Photos recorded during operation

- Six photos for field of lymph node dissection

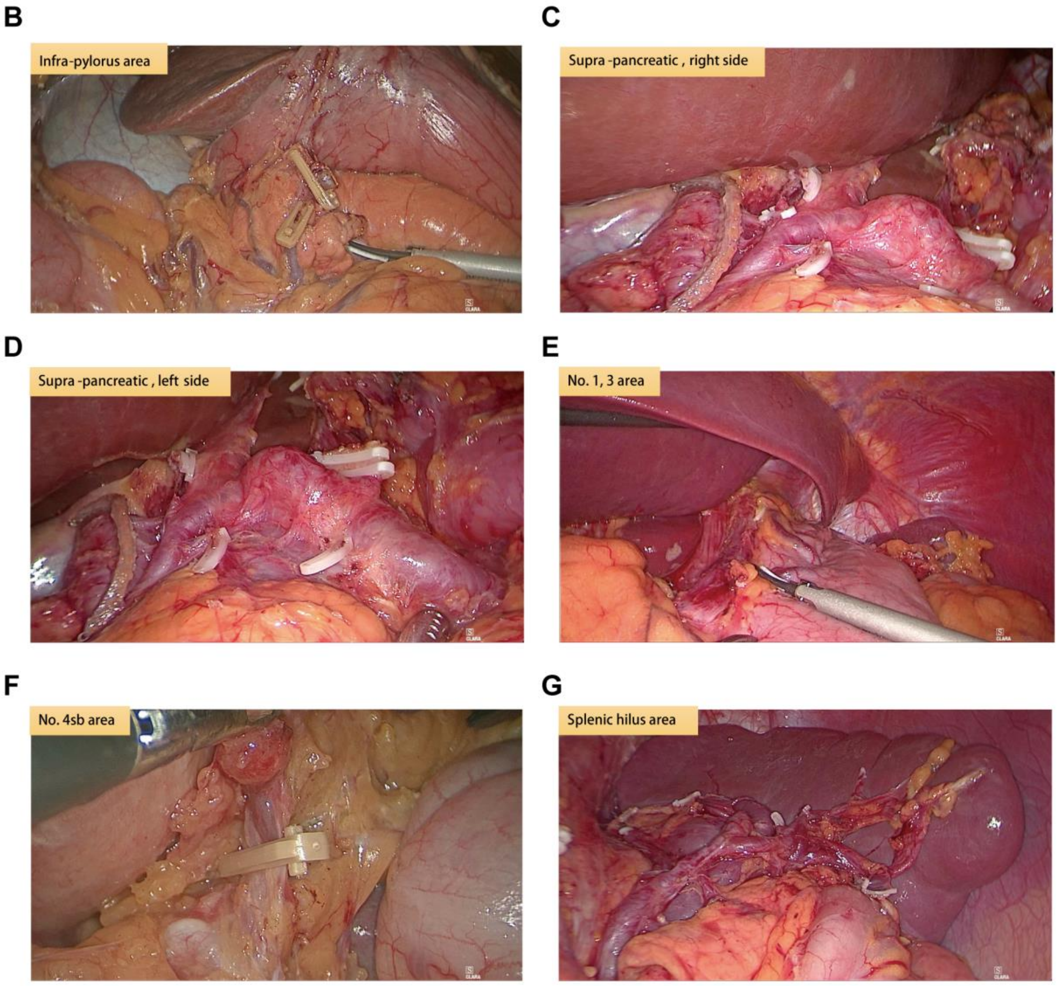

© 2021 Zheng C et al. *JAMA Network Open.*

eFigure 1. Surgical Quality Control for CLASS-04 Trial (A) and Field of Lymph Node Dissection (B-G)

(B) Infra-pylorus area; (C) Supra-pancreatic, right side; (D) Supra-pancreatic, left side; (E) No. 1, 3 area; (F) No. 4sb area; (G) Splenic hilus area.

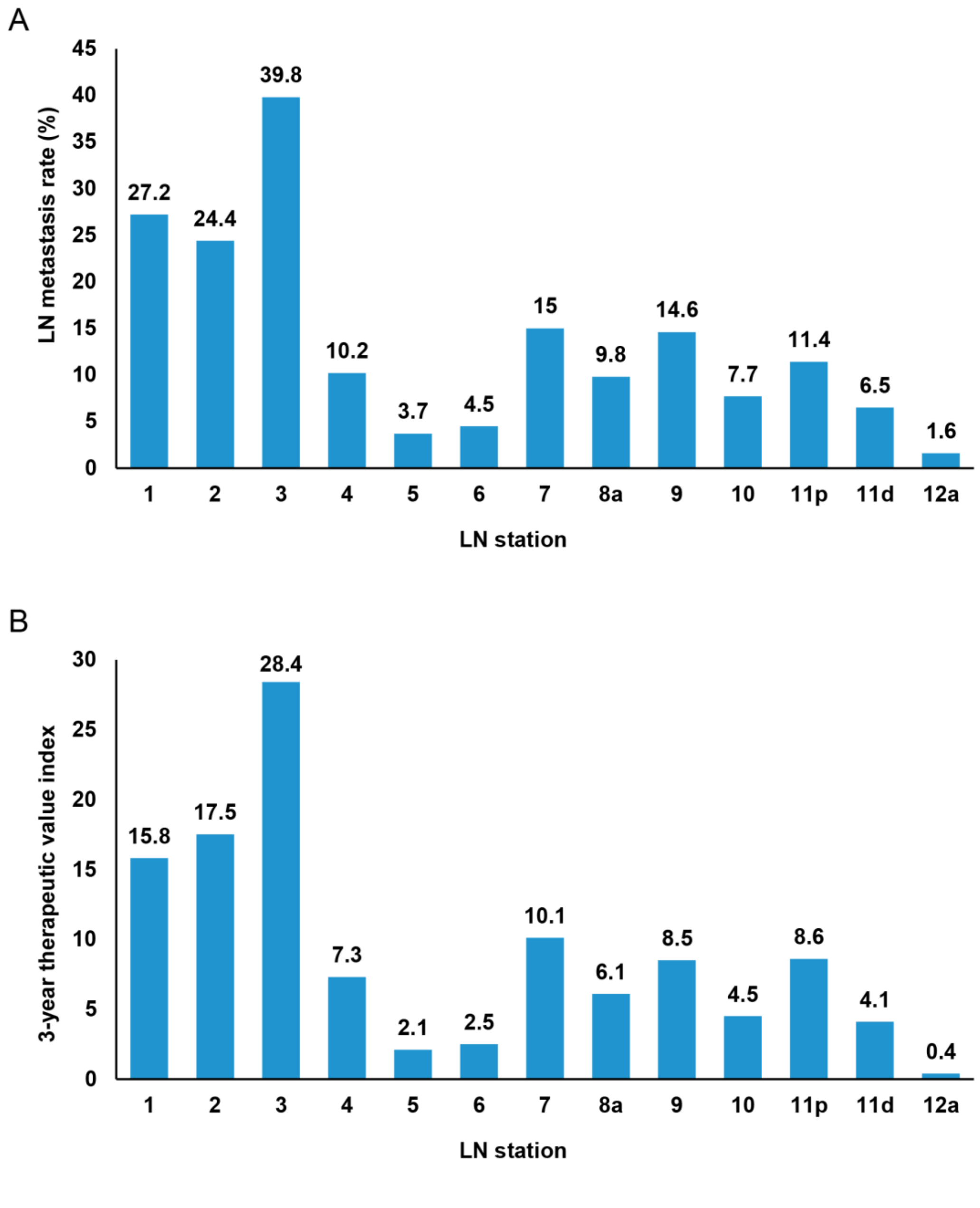

eFigure 2. The Incidence of Lymph Node (LN) Metastasis (A) and 3-Year Therapeutic Value Index (B) of LN Dissection

© 2021 Zheng C et al. *JAMA Network Open.*

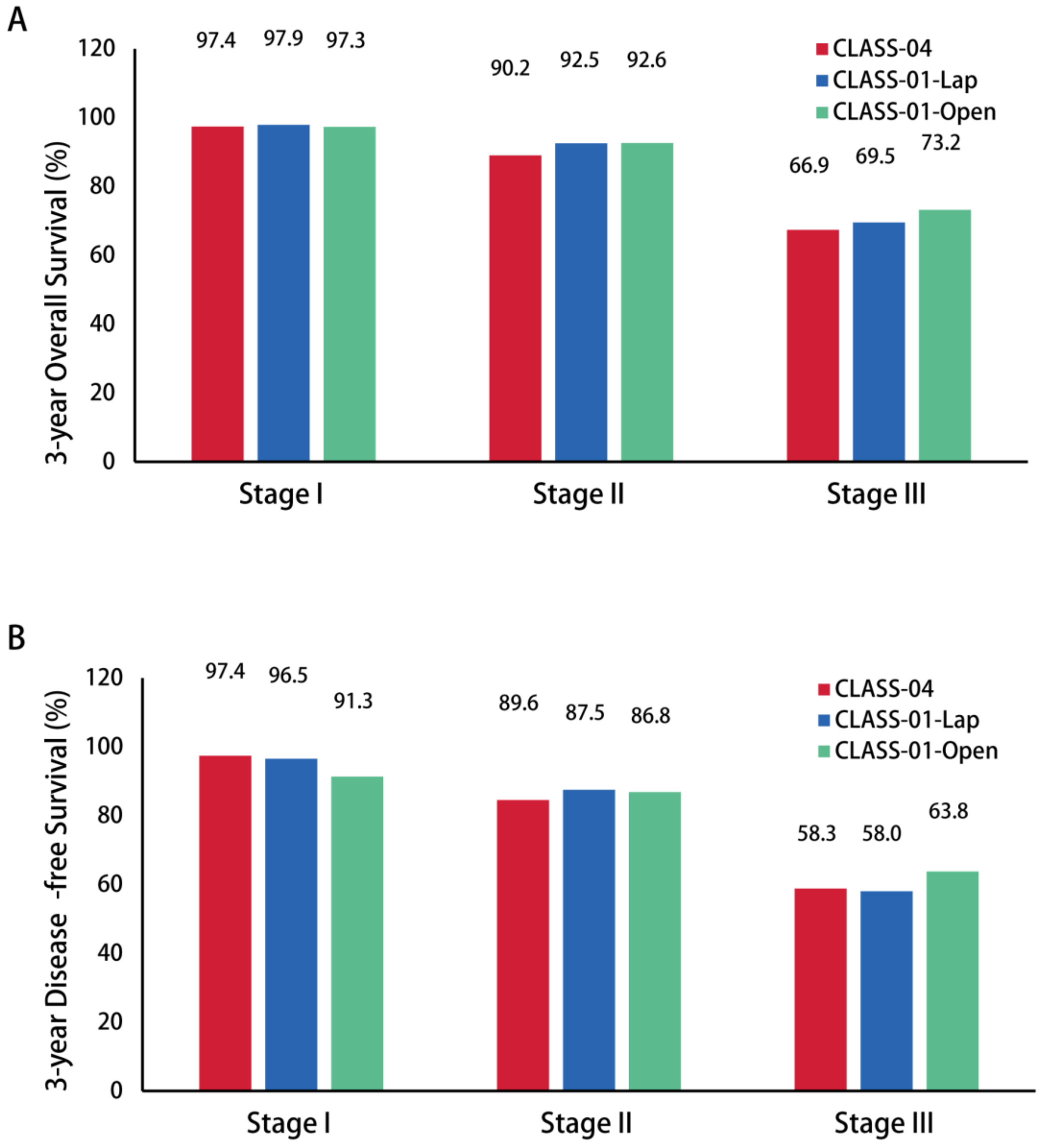

eFigure 3. Comparison of the long-term survival of patients with different stages in the CLASS-04 and CLASS-01 studies. Lap, Laparoscopic.

© 2021 Zheng C et al. *JAMA Network Open.*

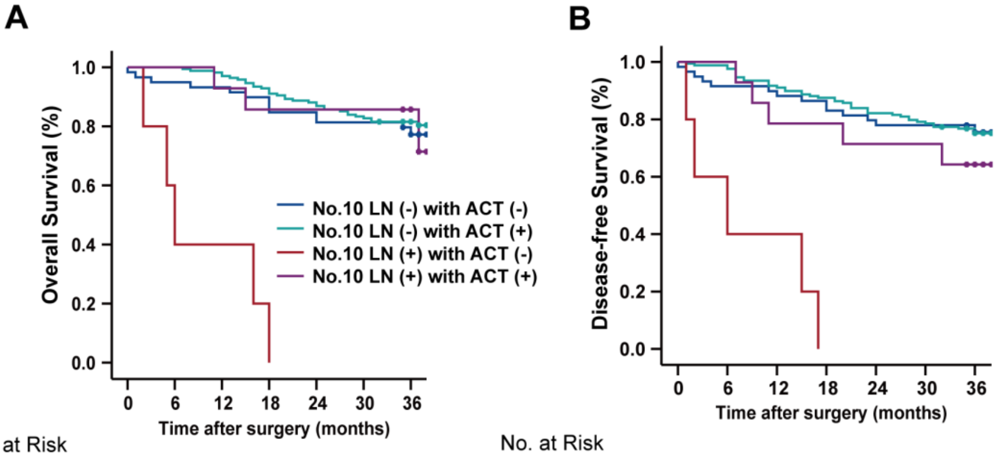

A

No. at Risk

	0	6	12	18	24	30	36
**No.10 LN (-)**							
**ACT (-)**	59	56	55	50	48	48	21
**ACT (+)**	168	168	163	153	146	139	70
**No.10 LN (+)**							
**ACT (-)**	5	2	2	0	0	0	0
**ACT (+)**	14	14	13	12	12	12	6

B

No. at Risk

	0	6	12	18	24	30	36
**No.10 LN (-)**							
**ACT (-)**	59	54	52	49	46	46	21
**ACT (+)**	168	164	153	147	138	132	67
**No.10 LN (+)**							
**ACT (-)**	5	2	2	0	0	0	0
**ACT (+)**	14	14	11	11	10	10	5

eFigure 4. Kaplan-Meier Curves of Overall Survival (A) and Disease-Free Survival (B) for Patients With No. 10 Lymph Node (LN) Metastasis and Without Metastasis Stratified by Adjuvant Chemotherapy

© 2021 Zheng C et al. *JAMA Network Open.*
